# The Ringed World: Saturn from Cassini to The James Webb Space Telescope

**DOI:** 10.1038/s41467-026-72081-9

**Published:** 2026-05-11

**Authors:** Paola Tiranti

**Affiliations:** School of Engineering, Physics and Mathematics, Northumbria University, Newcastle Upon Tyne, UK

**Keywords:** Giant planets, Space physics

## Abstract

Saturn’s rings actively supply material to the planet through charged and neutral inflows. The Cassini mission revealed this coupling and the James Webb Space Telescope now sharpens the picture, yet the variability, chemistry and future of this erosion remain unresolved.

## Saturn as a Coupled World

Saturn is the giant planet most immediately defined by its appearance: its broad rings bisect the body of the planet, making it unmistakable even in small telescopes. The rings are composed of water ice and dust, likely the debris of a disrupted icy moon^1^. For decades they were treated as a spectacular but separate feature, orbiting above the atmosphere and within the magnetosphere without shaping either.

The story that has emerged over the past decade is very different. The rings are not mere decorations but active participants. They feed the atmosphere. They imprint the ionosphere. They tug on the magnetosphere in ways that only now, with the full weight of Cassini’s data behind us, we can describe with confidence. That ring material falls into Saturn’s atmosphere, changes its chemistry, alters its conductivity, and links directly into the currents that shape the magnetosphere and the aurora. Saturn stops being “a planet with rings” and becomes a single, connected system.

## How the Rings Feed the Planet

The first hints of this connection came from Voyager^2^ and ground-based observations. Near-infrared Keck observations revealed that Saturn’s low- and mid-latitude ionosphere, expected to be smooth, instead showed a chain of bright ${{{\rm{H}}}}_{3}^{+}$ emission^3^. ${{{\rm{H}}}}_{3}^{+}$, one of the planet’s main ions emitting in the infrared, responds quickly to changes in temperature, composition and density, making it an excellent tracer of upper atmospheric structure. When the Keck features were mapped along magnetic field lines, they aligned with gaps and dense regions in the rings. This was a signature of “ring rain”: charged particles flowing along magnetic field lines into the atmosphere. Because Saturn’s magnetic field is offset, this process is strongly asymmetric, favouring the southern mid-latitudes and varying with season^2^.

Cassini’s Grand Finale^4^ revealed a second, very different process: “equatorial inflow”. This is material deriving from the rings, such as dust, molecules and nanograins particles, spiralling inward due to atmospheric drag as the rings interact with Saturn’s extended upper atmosphere^5^. Cassini demonstrated that much of this material is electrically charged. Two distinct dust populations were identified: an equatorial population dominated by water ice grains and a mid-latitude population enriched in silicates, both interacting with Saturn’s magnetic field but following different pathways^6^. This inflow was one of the major surprises of Cassini’s last orbits^6–9^.

These two forms of infalling material represent different physical regimes: magnetically guided inflow at mid-latitudes and drag-dominated inflow at the equator, even though dust grains in both regions may be electrically charged. Both modify Saturn’s upper atmosphere, but in different places and in different ways. A third contribution, from Enceladus, likely feeds both components. Its plumes supply much of the water influx that ultimately enters Saturn’s atmosphere^10^. Together, these processes form parallel flows linking Saturn’s rings, moons and atmosphere.

## The Atmospheric Response

Cassini’s final orbits showed that equatorial inflow is chemically rich: methane, water, ammonia, nitrogen, carbon-bearing molecules, and complex organics were all detected^11^. The associated nanograins sweep up electrons, creating dusty regions where electron densities fall and temperatures rise^12–16^. Numerical simulations show that the mid-latitude silicate population likely originates from interplanetary dust impacts that polluted the rings, with fragments subsequently transported along magnetic field lines into the atmosphere^6,17^. Meanwhile, the charged component of ring rain modifies mid-latitude ionospheric composition and contributes to the peaks identified in early ground-based ${{{\rm{H}}}}_{3}^{+}$ maps^18^.

An additional influence comes from the variable shadows cast by Saturn’s rings on its atmosphere. The opaque outer A and B rings block almost all EUV sunlight, while the inner C and D ring are more transparent^5,19^. These differences shape where photoionisation can occur, creating sharp contrasts in ionospheric density^20–23^. Essentially, the rings act as seasonally shifting shutters. It is therefore the combination of magnetically guided ring rain, drag-dominated equatorial inflow, and seasonal shadowing that sculpts Saturn’s atmosphere.

**Fig. 1:**
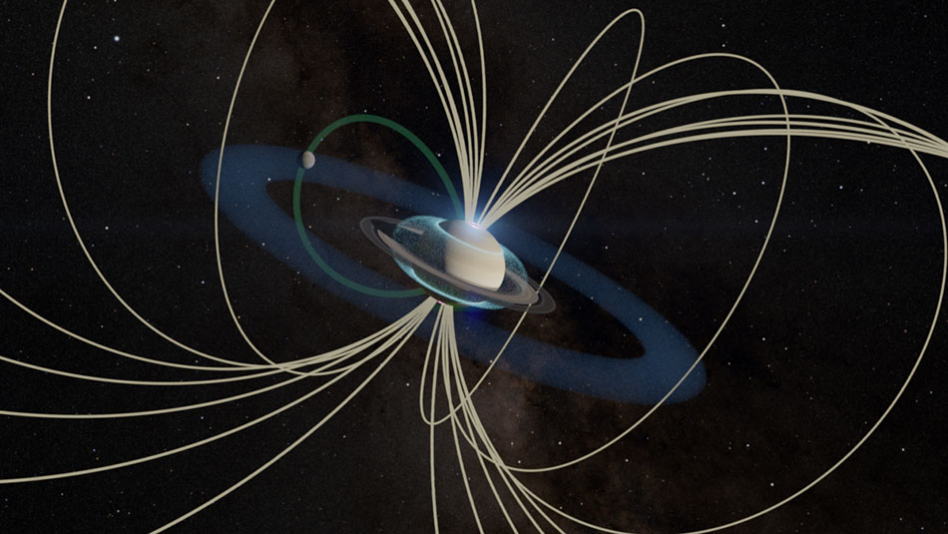
Illustration of Saturn’s coupled system during the January 2019 solstice. The main auroral oval is mapped along magnetic field lines far out in the magnetosphere, while the narrow band of ring rain appears much closer to the planet, just inside the main rings. Enceladus, not to scale, and its orbiting E ring are included, along with the footprint where plasma flow generated from the moon connects back to the atmosphere. Credit: 3D visualisation by James O'Donoghue (University of Reading). Saturn textures from Cassini imagery (NASA/JPL-Caltech/Space Science Institute). Magnetic field lines plotted using JupiterMag^32^.

The Cassini mission revolutionised how we think about the boundaries between Saturn’s regimes. As shown in Fig. 1, the picture is now clear: the rings are losing material^18,24^, that material falls into the atmosphere^12^, modifying atmospheric chemistry and conductivity^15,25^, currents thread through it^26^ and close back into the rings and magnetosphere.

## The Mystery of The Ring’s Age

There is a consequence from the inflowing material: if the rings are losing mass, they cannot last forever. Cassini measured the present-day inflow but could not reveal whether this rate has persisted over millions of years or represents a temporary phase. Models span a wide range of ring lifetimes, from tens of millions of years to values approaching the age of the Solar System^17,27,28^.

The question then is: are we simply lucky to live at a time when Saturn’s rings are so massive and bright? If today’s inflow were sustained, the main rings would erode on comparatively short timescales. In addition, we must consider that all of the giant planets have rings too, albeit much fainter. Do they experience similar mass loss or is Saturn’s system genuinely unique?

Answering these questions means monitoring how inflow varies with time, latitude and altitude, and how its volatile and dusty components behave^9^. It also means asking where ring-derived material shows up in the deeper atmosphere and in the stratosphere, and where it does not.

## The JWST Era: Seeing What Cassini Could Not

This is where the James Webb Space Telescope (JWST) is transformative. Many of the signatures of charged and neutral ring inflow are extremely faint in the infrared. Cassini lacked the sensitivity, and ground-based telescopes could only capture a glimpse of them.

The first JWST/Near-Infrared Spectrograph (NIRSpec) Integral Field Unit (IFU) observations of Saturn^29^ show that the mid-latitude ionosphere contains structured drifting “beads” of ${{{\rm{H}}}}_{3}^{+}$, likely shaped by thermospheric winds and current-modulated dynamics rather than ring-driven inputs. NIRSpec also detects an asymmetric methane fluorescence pattern in the upper stratosphere, revealing circulation that Cassini never resolved.

JWST/Mid-infrared Instrument (MIRI) observations^30^ complement this picture by mapping Saturn’s seasonal evolution, observing wavelengths inaccessible to Cassini. Despite Cassini’s detection of a volatile-rich inflow at the equator, MIRI finds no corresponding nitrile enhancements. This raises a key question: was Cassini’s equatorial inflow dominated by grains rather than vapour, or has the volatile input since 2017 diminished?

What is still missing is vertical information at higher altitudes. JWST sensitivity is therefore pivotal. Limb observations of ${{{\rm{H}}}}_{3}^{+}$ can provide the first direct temperature and ion density profiles at multiple latitudes and longitudes. These can tell us how energy is distributed with altitude, how the ionosphere cools, and how Saturn compares to Jupiter, where similar profiles have been obtained^31^. Deriving these profiles will connect JWST’s horizontal structures into a three-dimensional view of the upper atmosphere, revealing something that Cassini could not.

Most importantly, JWST provides the first realistic chance to detect equatorial ${{{\rm{H}}}}_{3}^{+}$ emission with the sensitivity needed to detect equatorial inflow remotely. Non-auroral ${{{\rm{H}}}}_{3}^{+}$ has been seen only twice from the ground, and never near the equator. If the equatorial inflow continues, JWST should detect its chemical and thermal effects. If it has weakened, JWST will show that too. Essentially, this is the only way to monitor ring mass loss until another spacecraft arrives.

## Solving The “Kronian Puzzle”

JWST’s capabilities open the door to long-term monitoring of Saturn’s atmosphere and rings, but they are only the beginning. Here, the “Kronian puzzle” refers to a simple but unresolved question: how do Saturn’s rings, atmosphere, and magnetosphere exchange material and energy over time, and what does that imply for the origin and lifetime of the rings? Cassini was foundational for at least one generation of planetary scientists, and its Grand Finale revealed that Saturn is not a set of isolated regions, but a single, dynamically coupled system. What remains unclear is how variable this coupling is and how it evolves over seasonal and geological timescales. A future Saturn orbiter could go further: resolve the variability of ring rain over time, probe deeper into the atmosphere, and measure currents and conductances in-situ with modern instrumentation.

In parallel, other missions and facilities will add essential context. Dragonfly will explore Titan’s surface and atmosphere, adding crucial context on organic chemistry and volatiles that impact the Saturn system. Proposed missions to Enceladus, now one of ESA’s top targets, aim to characterise this ocean world, whose habitability is shaped by its interaction with Saturn’s magnetosphere and plasma environment. Next-generation telescopes, from extremely large ground-based facilities to the Habitable Worlds Observatory, will extend what JWST has begun, both at Saturn and at exoplanets with debris discs and dense ring systems. This broader perspective aligns with current strategic priorities: both NASA’s Planetary Science Decadal Survey and ESA’s Voyage 2050 programme highlight the importance of planetary environments, ocean world systems, and the coupled evolution of atmospheres and magnetospheres.

If we can piece together Saturn’s circulation of material and energy we will do more than just solve the “Kronian puzzle”. We will learn how planets with debris behave, how long their rings can last, and how atmospheres respond when they are constantly fed from above. Saturn then becomes more than an iconic image. It becomes a benchmark for understanding how worlds evolve in complex space environments.
